# Residents and Anæsthetics

**Published:** 1907-06-15

**Authors:** 


### Resident Medical Officers' Department.

[Contributions to this Column are invited, and, if accepted, will be paid for.]

### RESIDENTS AND ANÆSTHETICS.

The article on "Fatal Anæsthetics" in a recent issue of The Hospital must have been read with special interest by resident medical officers. The facts stated therein and the principles involved demand the closest attention of every member of this branch of medical practice. The writer of the article adopts the judicial attitude. Having criticised the value of statistics and of hasty conclusions, he presents with much moderation the case for the administration of anæsthetics by house officers, and concludes with a qualified assent to Dr. Waldo's question, as to whether it is wise to allow men with less experience than special anæsthetists to do this work. This carefully weighed expression of opinion does not, however, close the subject. Before very long, in the ordinary course of events, further misadventures during the administration of anæsthetics in hospital will occur, and it is probable that they will be attended with even greater publicity than were those which formed the subject of this inquiry. Public interest, once excited on a matter of this kind, is with difficulty restrained. Guy's Hospital is at present the scape-goat; unless we are mistaken, it will soon be the turn for some other teaching hospital to attain an unenviable notoriety in this respect.

The British public does not give voluntary support to its hospitals without expecting some *quid pro quo* in the form of control over their methods of administration. In former times this mental attitude was confined to the educated classes, the subscribers of large sums of money. Nowadays it is shared by the lower classes, the givers of pence, whose heads have been turned by the democratic eloquence of the halfpenny press, and by the two-edged triumph of the Labour party at the last General Election. This latter class, always responsive to any appeal to its passions, always unmoved by appeals to its reason, has already begun to feel the lust of power. It will soon become a factor to be reckoned with by the governors of our hospitals, and one whose influence will not be for its own ultimate benefit, or for the good of the community in general.

Conscious of these social changes which are already at work in England, we foresee endless complications which may arise from them in the administration of hospitals. A few more deaths under anæsthetics, investigated at the coroner's court, and shown to the public gaze by the fierce search-light of the Press, may conceivably force the hands of hospital authorities, and result in the withdrawal of anæsthesia from the domain of the house officer. Nothing more prejudicial to the public welfare than this could possibly be imagined. Some of the disadvantages, both to the practitioner and to his patients, which would arise from the withdrawal of this privilege have already been indicated by the author of the article in The Hospital of June 8. We would urge every reader of this section to bear this most undesirable possibility in mind, and to consider for himself the consequences—immediate and remote, individual and collective—of the restriction of anæsthetics to those only who have made a speciality of the subject. It is the duty of every student, and of every house officer, general practitioner, and consultant, to resist to the utmost of his power any attempt to interfere with the right of the qualified medical practitioner to administer anæsthetics, whether in public institutions or in private. Specialisation has gone quite far enough already. Originating within the profession, it cannot be helped; but it must not be forced upon us by the public, acting through the coroner, the halfpenny press, and the weaker-kneed members of the hospital governing boards.

Finally, we must admit that the publicity which has recently been given to deaths under anæsthetics will certainly do good in one direction at least. It cannot but act as an excellent stimulus to the medical schools and examining boards of this country. It will unquestionably stimulate the medical schools to improve their courses of instruction in this branch of practice, and to increase the facilities for students to acquire a thorough practical acquaintance with the administration of anæsthetics. The regulations for admission to the Final Professional Examinations of the various licensing bodies will no doubt soon be so extended that it will be impossible for anyone to obtain a qualification to practise without first undergoing a thorough practical training in the induction and maintenance of anæsthesia by all the recognised methods. At present it is still possible, in certain hospitals, for a student to be "signed up" for his "Finals" with nothing more than a theoretical acquaintance with anæsthetics; and it is equally possible for him to emerge successfully from his examinations without having had to answer a single question on this most important subject.

### ST. THOMAS'S HOSPITAL.

House Appointments.—The following gentlemen have been selected as house officers at St. Thomas's Hospital from Tuesday, June 4, 1907:—Casualty officers (from July 1, 1907): (Senior) A. B. Howitt, (Junior) W. O. Sankey. Resident house physicians: E. V. Dunkley (Extension), H. A. Philpot (Extension), C. E. Whitehead, H. G. Bennett. House physicians to out-patients: G. G. Butler (Extension), S. L. Walker (Extension), W. H. R. Sutton, S. Churchill. Resident house surgeons: C. M. Page (Extension), S. G. MacDonald (Extension), H. B. Whitehouse (Extension), R. L. Gamlen (Extension). House surgeons to out-patients: H. J. Nightingale (Extension), H. R. Unwin (Extension), G. M. Huggins (Extension), F. M. Neild (Extension). Obstetric house physicians (Senior) A. C. D. Firth, (Junior) F. S. Hewett. Ophthalmic house surgeons: (Senior) W. C. A. Ward (Extension), (Junior) A. S. Burgess (Extension). Special departments: (Throat) W. R. Bristow (Extension), R. E. Todd; (skin) A. J. S. Pinchin, E. A. Sparrow; (ear) A. J. S. Pinchin, H. E. T. Dawes. Children's surgical: H. H. Carleton (Extension), R. E. Todd. Electrical Department, *x*-ray Department: A. J. H. Iles.

